# Predictors and Mediators of Outcome of a Focussed, Programme Led Intervention for Young People With Eating Disorders

**DOI:** 10.1002/eat.24516

**Published:** 2025-08-11

**Authors:** Daniel Wilson, Renata A. Mendes, Natalie J. Loxton

**Affiliations:** ^1^ Children's Health Queensland Hospital and Health Service Child and Youth Mental Health Service, Eating Disorders Team Brisbane Australia; ^2^ Child Health Research Centre University of Queensland Brisbane Australia; ^3^ School of Applied Psychology Griffith University Brisbane Australia

**Keywords:** adolescent, anorexia nervosa, focussed and programme led interventions

## Abstract

**Background:**

Focussed and programme‐led interventions have been of increased interest in the eating disorders (EDs) field as a potential solution to barriers in accessing timely, effective treatments. Little is known about the mechanisms through which these treatments work or for whom they are most suitable.

**Objective:**

To identify mediators and predictors of outcome in a focused, program‐led intervention for young people with EDs.

**Method:**

Participants included young people with an ED (*n* = 169, female = 92.9%, M_age_ = 14.46 years) and their parents/caregivers (female = 147, male = 92), who undertook a 6‐week parent‐focused, psychoeducation‐based treatment program and completed measures pre and post treatment. Young person measures included BMI centile, self‐report measures of dietary restraint, ED‐related obsessions and rituals, and expressed emotion (perceived criticism), and parents completed measures of self‐efficacy.

**Results:**

Overall, the models explained modest variance in outcomes. Results showed that increased parental self‐efficacy mediated the effect of the treatment on reductions to dietary restraint, but not for BMI centile. Perceived criticism predicted the effect of the treatment on BMI centile, such that higher levels of perceived criticism were associated with less weight gain. ED‐related obsessions and rituals predicted the effect of the treatment on dietary restraint, although the high correlation between these measures may limit the meaningfulness of this result.

**Discussion:**

The findings support existing research in traditional treatments highlighting the benefits of empowering parental self‐efficacy to progress ED recovery; it highlights the need for supporting families with high expressed emotion.

**Summary**

- Parental self‐efficacy mediated the effect on dietary restraint of a six‐week program for parents of young people with ED.
- The mediation model was not supported for BMI Centile.
- Parental expressed emotion predicted the effect of the treatment on BMI Centile, with higher perceived criticism resulting in less weight gain.

Eating disorders (EDs) can be severe and chronic conditions and are associated with comorbid psychological and physical sequelae, as well as significant impairments to functioning and socioeconomic burden (GDB Mental Disorders Collaborators 2022; Sawyer et al. 2016; Wilson et al. 2025). The onset of ED typically occurs during adolescence, making timely intervention at this stage vital, as prolonged illness and delayed access to treatment are predictors of poor prognosis (Carter et al. 2012; Forman et al. 2011; Von Holle et al. 2008). Traditional models of treatment typically involve prolonged courses of treatment with highly specialized and well‐trained practitioners (Kazdin et al. 2017). However, recent increases in prevalence and associated demands to service provision mean that access to timely intervention with existing models of treatment is not always possible (Viljoen et al. 2024).

In this context, there has been recent interest in alternate treatment approaches which aim to increase accessibility to early and effective intervention, with particular focus on programme led (i.e., where rather than with the clinician, the expertise lies with the programme, for example a book, app or computer program), and focused interventions (i.e., an intervention that takes less than 50% of clinician time compared to traditional interventions; see Davey et al. 2023 for a detailed overview). Although the evaluation of such treatments is mostly preliminary, they show promise. For example, programme led and focused versions of Cognitive Behavior Therapy (CBT) for EDs show comparable results to the more traditional and well‐established enhanced cognitive behavior therapy (CBT‐E) in non‐underweight adolescents/young adults with EDs (Allen et al. 2024). Additionally, there have been several interventions designed to support parents waitlisted for other treatments (e.g., Family Based Treatment; FBT), including videos and reading materials (Couturier et al. 2023), guided self‐help (Wade et al. 2022), workshop series and medical monitoring (Sidari et al. 2025), and single seminar sessions with follow‐up telephone support (Spettigue et al. 2015). Results from these interventions have shown weight gain and improved parental self‐efficacy (Couturier et al. 2023; Sidari et al. 2025; Wade et al. 2022). Additionally, while Spettigue et al. (2015) found no change in ED symptoms, Wade et al. (2022) and Sidari et al. (2025) reported decreases in ED symptoms and behaviors, with Sidari et al. (2025) also reporting decreases in depressive symptoms among young people.

These studies suggest that programme‐led, focused interventions may have utility within the spectrum of treatments for individuals and families experiencing EDs. However, given the relatively early stage of evidence supporting such interventions, further research is needed to clarify their mechanisms of action and to determine which individuals are most likely to benefit (Davey et al. 2023). Identifying mediators could help improve the effectiveness of such interventions as well as guide the removal of redundant/superfluous elements, while identifying predictors of outcome may further inform personalization and treatment matching (Davey et al. 2023). To date, no study to our knowledge has investigated mediators or predictors of outcome in programme‐led, focused ED interventions for adolescents.

## 1 The Current Study

The current study aimed to identify mediators and predictors of a programme led, focused intervention—Strong Foundations (Sidari et al. 2025). The intervention involves 6 weekly psychoeducation seminars and brief specialist medical monitoring sessions, with the primary goal of the intervention being to increase parents' understanding of EDs and improve parental self‐efficacy in treating their child's ED. It was introduced to a specialist child and adolescent EDs clinic to increase support to families whilst awaiting more traditional evidence‐based treatments. The programme has been shown to improve young person's ED symptoms (*d* = 0.32), depressive symptoms (*d* = 0.28) and BMI centile (*d* = 0.27), as well as improve parental self‐efficacy (*d* = 0.47) and depressive symptoms (*d* = 0.17; Sidari et al. 2025).

For the outcome variables of interest, we focused on dietary restraint and BMI centile, as improvement to both of these markers has been shown to be predictive of positive outcomes in evidence‐based treatments for people with restrictive EDs (Le Grange et al. 2014; Turner et al. 2015). Dietary restraint was chosen as an outcome variable rather than a broader measure of ED psychopathology (i.e., that also captures shape and weight concerns), as Strong Foundations targets dietary restraint and underweight status specifically (i.e., through nutritional, meal support and distress tolerance psychoeducation) rather than body image or shape and weight concerns more broadly (NOTE: results for the main analyses including broader ED psychopathology are included in the supplemental materials).

As to the potential mediators or mechanisms of action, it has been posited that exposure to the Strong Foundations intervention improves parental self‐efficacy, which better equips parents to notice and circumvent ED behaviors in their children (Sidari et al. 2025). As such, it was hypothesized that the effect of Strong Foundations on dietary restraint and BMI centile would be mediated through improvements to parental self‐efficacy.

Given the parental focus of Strong Foundations, potential predictors of treatment effectiveness were drawn from previously identified predictors and moderators of FBT (Hamadi and Holliday 2020). Expressed emotion has been identified as a construct of influence in the treatment of adolescent anorexia nervosa (AN). Expressed emotion refers to a family member's behavior and interactions with their unwell relative and is comprised of the facets of criticism, hostility, emotional overinvolvement, positivity, and warmth (Brown et al. 1972). Studies have found maternal criticism, in particular, is associated with poorer treatment outcomes (e.g., Eisler et al. 2000, 2007; Rienecke et al. 2016). Similarly, ED‐related obsessions and rituals are related to treatment outcome, with findings indicating that those with higher ED obsessions and rituals require a longer course of FBT (e.g., Lock et al. 2005), do less well in individual compared to family treatments (e.g., Le Grange et al. 2012), and are less likely to remit from ED behaviors post‐treatment (e.g., Le Grange et al. 2015). Consequently, expressed emotion (perceived criticism specifically) and ED‐related obsessions and rituals were hypothesized to predict the effect of Strong Foundations on changes to dietary restraint and BMI centile (Lock and Le Grange 2019). It was expected that higher levels of both predictor variables would be associated with a less effective intervention.

## 2 Method

### 2.1 Participants

The study involved 169 participants (female = 92.9%), with an average age of 14.46 years (SD = 1.59, range 9–17). Caregivers consisted of female caregivers (*n* = 147, M_age_ = 47.14 years, SD = 4.75) and male caregivers (*n* = 92, M_age_ = 49.03 years, SD = 5.39), referred to herein as parents for simplicity. Participants were recruited from consecutive referrals of young people commencing treatment at a public outpatient service specializing in child and youth EDs. For inclusion into the study, young people had to meet International Classification of Diseases and Related Health Problems (10th ed.; ICD‐10; WHO, 2016) criteria for AN (81.7%) or Atypical Anorexia Nervosa (AAN; 18.3%), meet the clinic's suitability criteria (below 18 years of age; primary diagnosis of an ED; medically and psychologically stable for outpatient treatment; and consent/assent to treatment at the clinic), and consent for their data to be used for research. Participants were initially evaluated by a psychiatrist and a multidisciplinary team with experience in diagnosing and treating EDs in children and adolescents. Participants completed measures as part of routine clinical practice using tablet devices at initial assessment (pre‐treatment) and after completing the 6‐week Strong Foundations program. If parents consented to their data being used for research purposes, they were included in the study. Families completed the program while awaiting allocation to traditional outpatient ED treatments (e.g., FBT, CBT‐E), which they transitioned to after completion of Strong Foundations.

### 2.2 Procedure

Strong Foundations is a 6‐week, psychoeducation‐based, program led, focused intervention that was developed to improve parents' understanding of EDs and, in doing so, increase parental self‐efficacy in treating the ED (Caldwell et al. 2023). The program was designed to increase parental self‐efficacy and aimed to facilitate early changes in disrupted eating patterns for families awaiting traditional treatment models (e.g., FBT). The intervention comprises two main components: psychoeducation seminars and specialist medical management and support.

Parents attend a weekly online psychoeducation seminar delivered by a clinician experienced in adolescent EDs. Psychoeducation has been posited to have an integral role in improving parental self‐efficacy (Gorrell et al. 2022). The seminars cover topics relevant to EDs, consisting of general psychoeducation, nutrition, meal support, distress tolerance, and communication, treatment of EDs, and support services. There are six total seminar sessions held over 6 weeks, as shown in Table 1.

**TABLE 1:** Seminar topics and content for each weekly Strong Foundations session, also described in Sidari et al. (2025).

Seminar topic	Content covered
General ED psychoeducation	Types of EDs, common warning signs and behaviors, starvation syndrome and the Minnesota starvation experiment, refeeding syndrome, importance of medical monitoring, hospital admission guidelines
Nutrition	Dieting as an ED risk factor, adolescent nutrition for growth and development, nutrition required for recovery or weight restoration, physical changes during weight restoration, practical guide to energy density, nutritional complexities (e.g., allergies, intolerances, nutrient deficiencies)
Meal support	Rational, importance during re‐feeding, stages, helpful statements, externalization of the ED, reflective activities
Distress tolerance and emotional support	Distress tolerance and ED, helpful communication techniques, emotion coaching (e.g., validation, emotional support), emotion psychoeducation, emotional regulation techniques, safety planning
ED treatment	Medical monitoring—rationale and components, ED myths and misconceptions, carer distress and supports, evidence‐based ED treatments, FBT, CBT‐E, managing co‐morbidities, role of medications
Carer supports	Sharing recovery stories, useful carer support resources and information (e.g., national helplines, support organizations, support groups, workshops), how to access further supports

Families also attend a brief (30–45 min) weekly face‐to‐face specialist medical management and support appointment. These sessions focus on reviewing medical status, risk status, and medication, as well as providing containment and supporting basic meal support and refeeding principles in line with FBT (Lock and Le Grange 2012), as shown in Table 2. Full details of the Strong Foundations interventions are provided in Sidari et al. (2025).

**TABLE 2:** Focus for medical monitoring sessions.

Focus	Description
Review of medical status	Review of medication observations from young person's general practitioner, repeat observations if indicated to determine suitability for ongoing community management. Facilitation of hospital admission in conjunction with GP if indicated using the Queensland Child and Adolescent State‐wide guidelines
Risk management	Mental state assessed, risk assessment and management if required relating to suicidal ideation, self‐harm behaviors/ideation, aggression and caregiver fatigue/burnout
Medication review	Review of current medication or commence prescription if indicated for symptomology (e.g., mood/anxiety difficulties, sleep)
Containment	Providing validation, empathy and distress tolerance techniques if required to families and young people
Supporting re‐feeding	FBT‐informed guidance and support for re‐feeding and weight restoration
Consultation liaison	Liaison with other. Involved health professionals, school or work supports

### 2.3 Measures

#### 2.3.1 Demographic and Clinical Variables

Young people and parents/carers responded to items relating to their age and sex. Psychiatric diagnoses, weight, and height were collected by the assessing clinician.

#### 2.3.2 Body Mass Index (BMI) Centiles

BMI centiles were calculated using the young person's weight, height, and the Centre for Disease Control and Prevention growth charts (www.cdc.gov/growthcharts). Measurements were taken at the start and end of treatment.

#### 2.3.3 Parents Versus Anorexia (PVA)

The PVA is a 7‐item scale that measures parental self‐efficacy, which is conceptualized as parents' belief they have the ability to adopt a primary role in treating their child's ED (Rhodes et al. 2005). It has demonstrated acceptable reliability (*α* = 0.78) in previous studies (Rhodes et al. 2005). This measure was completed at the start and end of treatment.

#### 2.3.4 Eating Disorder Questionnaire Adolescent (EDE‐A)

EDE‐A is a modified version of the Eating Disorder Examination Questionnaire (EDE‐Q; Fairburn and Beglin 1994), with developmental modifications by shortening reference time frames from 28 to 14 days and simplifying some wording (Carter et al. 2012). For the purposes of this study, we utilized the restraint subscale only, which captures dietary restraint on a 0–6 Likert scale, with a higher score representing greater restraint. This measure was completed at the start and end of treatment.

#### 2.3.5 Brief Dyadic Scale of Expressed Emotion (BDSEE)

The perceived criticism subscale of the BDSEE (Medina‐Pradas et al. 2011) was used to assess how critical the young person perceived their primary caregiver to be toward them. The subscale includes four items, with scores ranging from 4 to 40; higher scores indicate greater perceived criticism. Young people responded to the prompts relating to their mother if she was a primary caregiver. If their mother was not a primary caregiver, they responded relating to whoever this was. The scale has demonstrated acceptable reliability in previous studies (*α* = 0.73–0.90; Wilson et al. 2025). Only the perceived criticism subscale was used in this study, as this aspect of expressed emotion has been shown to influence FBT outcomes (e.g., Eisler et al. 2000, 2007; Rienecke et al. 2016). This measure was completed at the start of treatment only.

#### 2.3.6 Yale‐Brown‐Cornell Eating Disorder Scale—Self‐Report (YBC‐EDS‐SR)

The YBC‐EDS‐SR (Fitzpatrick and Weltzin 2014) is a 17‐item self‐report questionnaire that measures obsessions and compulsions related to eating. The 8‐item global subscale was used for this study, which measures total ED obsessions and rituals. This subscale has demonstrated excellent internal reliability (*α* = 0.92; Wilson et al. 2025). This measure was completed at the start of treatment only.

### 2.4 Ethics

The project was approved by the Children's Health Queensland research ethics committee (HREC/20/QCHQ/67708), with consent obtained from parents/legal guardians as per ethical approval.

### 2.5 Statistical Analysis

All analyses were conducted using SPSS version 30. Relationships between the baseline variables were investigated using bivariate correlations. Mediation effects were tested using model 1 of the MEMORE macro, which allows estimation of the indirect effect of mediators in within‐subject/repeated measures design by constructing the difference between repeated mediator and dependent variables measurements in accordance with Montoya and Hayes (2017) and Judd et al. (2001). Two mediation models were tested, including time (i.e., the intervention) as the independent variable, and PvA at the start and end of treatment as the mediator variable. PvA was a score from one “primar” parent using the following logic: if there was only one parent who attended and completed the measures at both time points, they were the primary parent; in the case of both parents providing responses at each time point, the default was for the female to be the primary parent to be more consistent with previous research that has exclusively focused on maternal variables (e.g., Eisler et al. 2000, 2007; Rienecke et al. 2016). (NOTE: The main analysis was run using female parents only, with no changes to the interpretation of the results). Restraint and BMI Centile at the start and end of treatment were dependent variables for each analysis. Indirect (i.e., mediated) effects were tested using 95% confidence intervals based on 10,000 samples. Significant effects are indicated by an absence of zero within the confidence intervals.

Two linear regression analyses were run with ED obsessions and rituals, and the BDSEE perceived criticism subscale as predictors; change in Restraint and BMI Centile scores (pre‐post SF) as dependent variables.

### 2.6 Missing Data

The dataset consisted of multiple informants (young person, either or both parent) over multiple time points, which led to significant missing data, especially at the post‐treatment collection time points for restraint (33.34%) and the PvA (35.54%) subscales. Little's MCAR test showed that the pattern of missingness was missing completely at random (chi‐squared = 39.41, df = 36, *p* = 0.32). Further, we conducted a logistic regression analysis with missingness at post‐treatment predicted by baseline value. Those with higher food‐related obsessions and rituals were more likely to have end‐of‐treatment data. This is consistent with data missing at random, as missingness depends on an observed variable. However, it does not rule out the possibility that the data are missing not at random. The implications for the interpretation of the results may mean that they are less generalizable to young people with less severe food‐related obsessions and rituals; however, the results may be strengthened by a bias toward a more severe cohort, therefore being less likely to overestimate treatment effects.

Intention to treat analyses were used to account for young people and parents with missing data at the end of Strong Foundations, whereby their initial scores were entered as their end of treatment score (i.e., an effect size of zero). The interpretation of the mediation and regression analyses held consistent when listwise deletion for those with missing data at post treatment was applied. Young people with missing data for baseline variables were deleted listwise.

## 3 Results

### 3.1 Baseline Characteristics

Means and bivariate correlations between the baseline variables are shown in Table 3.

**TABLE 3:** Means, standard deviations, and baseline bivariate correlations.

Measure (range)	Pre mean (SD)	Post mean (SD)	1	2	3	4
1. BMI centile	36.87 (28.48)	44.48 (27.33)	—			
2. Restraint (0–6)	3.37 (1.99)	2.68 (1.98)	0.11	—		
3. ED obsessions and rituals (0–32)	15.80 (7.36)		0.01	0.62*	—	
4. Perceived criticism (4–40)	18.62 (8.09)		0.20*	0.15*	0.21*	—
5. Self‐efficacy (5–35)	18.98 (3.68)	20.62 (3.91)	−0.03	−0.06	0.10	−0.08

*Note*: *p < 0.05. ED obsessions and rituals = Yale Brown Cornell eating disorders scale; perceived criticism = brief dyadic scale of expressed emotion—perceived criticism subscale; self‐efficacy = parent versus anorexia scale.

### 3.2 Mediation Analysis

The results of the first mediation model showed that changes to parental self‐efficacy were found to significantly mediate the effects of time (intervention) on the reduction of Restraint scores (indirect effect = 0.21, SE = 0.09, 95CI: 0.05; 0.41). The total model explained 6% of the variance in changes to restraint scores (*R* ^*2*^ = 0.06, *F* (2,159) = 5.09, *p* < 0.01).

Changes to parental self‐efficacy did not significantly mediate the effect of time on increases to BMI centile, and the total model did not explain significant variance in changes to BMI centile (*R* ^*2*^ = 0.01, *F* (2,162) = 0.50, *p* = 0.61).

### 3.3 Regression Analysis

The first multiple regression analysis was conducted with ED obsessions and rituals and perceived criticism as predictors of change in Restraint. The model was found to be statistically significant, *R* = 0.26, *R* ^*2*^ = 0.07, *F* (2, 119) = 4.32, *p* = 0.015, explaining 7% of the variance in change in Restraint. ED obsessions and rituals were a significant predictor, while perceived criticism was not significant, as shown in Table 4. The magnitude of the effect was that greater baseline levels of ED obsessions and rituals were associated with greater reductions in restraint scores across the intervention. To test the effect of weight status on improvements to restraint, two analyses were run. In the first, baseline BMI centile was included as step one of the regression for change in restraint scores and was not a significant predictor (B = −0.12, SE = 0.56, *t* = −0.84, *p* = 0.84), and there was no change to the interpretation of the main predictors when including baseline BMI centile as a covariate. We also ran a model including underweight status operationalized as binary above/below 5th centile (Sidari et al. 2025; Zanna et al. 2021) as an interaction term and found no evidence of moderation on change in restraint scores (F (1, 161) = 0.25, *p* = 0.62).

**TABLE 4:** Effects of ED obsession and rituals, and perceived criticism on changes to restraint and BMI centile across strong foundations.

Criterion	Variable	B	SE	*p*	95% CI lower	95% CI upper	sr^2^
Change in restraint							
	ED obsessions and rituals	0.064	0.022	< 0.01	0.02	0.11	0.068
	Perceived criticism	−0.011	0.020	0.57	−0.05	0.03	0.002
Change in BMI centile							
	ED obsessions and rituals	0.001	0.002	0.46	−0.00	0.01	0.001
	Perceived criticism	−0.004	0.002	0.01	−0.01	−0.00	0.053

*Note*: Unstandardized coefficients are shown. CI = confidence interval. ED obsessions and rituals = Yale Brown Cornell eating disorders Scale; perceived criticism = brief dyadic scale of expressed emotion—perceived criticism subscale. Change in restraint = pre score‐post score, meaning that a higher score indicates greater reductions in dietary restraint across the treatment. Change in BMI centile = post score‐pre score, meaning that a higher score indicates more weight gain across the treatment. As such, positive coefficients predict a greater response to treatment, whereas negative coefficients predict a lesser response to treatment.

The second multiple regression analysis was conducted with ED obsessions and rituals and perceived criticism as predictors of change in BMI centile. The model was found to be statistically significant, *R* = 0.23, *R* ^*2*^ = 0.05, *F* (2, 119) = 3.38; *p* = 0.04, explaining 5% of the variance in BMI centile change. ED obsessions and rituals were not a significant predictor, whereas perceived criticism was a significant predictor of change in BMI centile, as shown in Table 4. The effect of Strong Foundations on increasing BMI centile was reduced for greater levels of perceived criticism.

## 4 Discussion

This study was the first to investigate the indirect effects of a focused, program‐led intervention on important outcome variables for adolescent EDs. We found that baseline restraint scores were positively associated with baseline ED obsessions and rituals and perceived criticism. Positive associations were also found between perceived parental criticism and BMI centile and ED obsessions and rituals. Together, these associations support previous research highlighting the relevance of expressed emotion and ED obsessions and rituals in ED symptoms and treatment (Hamadi and Holliday 2020; Rienecke et al. 2016; Rienecke and Le Grange 2022).

The mediation analysis supported parental self‐efficacy as a mechanism through which the Strong Foundations program was effective in reducing dietary restraint. This supports the notion by Sidari et al. (2025), who posited that Strong Foundations may have increased knowledge and skills among parents, which in turn may have improved their self‐efficacy to better enable them to support their child and circumvent ED behaviors. This is consistent with the main targets of FBT, which is to empower families to restore eating patterns that the ED may have disrupted (Lock and Le Grange 2012).

The mediation analysis did not support improved self‐efficacy as an indirect pathway through which Strong Foundations led to increased BMI centile, suggesting that other mechanisms may be influencing weight gain across Strong Foundations. It may also be that the timeframe of the outcome variables (i.e., 6 weeks) was not long enough to capture the effect of improved parental self‐efficacy on weight gain. Although not possible methodologically in the setting of the current study (as most participants continue to another treatment, e.g., FBT), future research could explore the same analysis with a longer follow‐up period.

The regression analyses investigated if ED obsessions and rituals and perceived criticism predicted the effectiveness of Strong Foundations. For dietary restraint change, ED obsessions and rituals predicted the effect of Strong Foundations, such that the intervention had a greater effect for those who scored higher on baseline measures of ED obsessions and rituals. This was the opposite direction to expected results and inconsistent with previous research showing that increased levels of ED obsessions and rituals present a barrier to ED treatment (e.g., Le Grange et al. 2015; Lock et al. 2005). However, it was consistent with results indicating that a family‐involved approach (as in Strong Foundations) is more effective than an individual approach for those with high eating‐related obsessionally (e.g., Le Grange et al. 2012). One possible explanation is the difference in outcome variables: this study measured dietary restraint, whereas previous studies have focused on remission from ED, defined as achieving 95% of Ideal Body Weight and EDE‐Q global scores within one standard deviation of community norms (e.g., Le Grange et al. 2012). In the current study, dietary restraint was highly correlated with ED obsessions and rituals (as assessed by the YBC‐EDS), indicating the scales may be measuring very similar constructs. Consequently, it would be expected that those with high YBC‐EDS scores would also have high restraint scores, such that these individuals had high potential to show reductions. Those with low YBC‐EDS scores would also be more likely to have low restraint scores and have less potential to show reductions across treatment (floor effect). Thus, the effect found may not be clinically meaningful.

For BMI Centile change, ED obsessions and rituals were not significant predictors in the model, whereas perceived criticism predicted the effectiveness of Strong Foundations, such that young people who perceived their primary caregiver to be more critical of them were less likely to gain weight across treatment. This is consistent with the FBT literature, which highlights that high levels of expressed emotion are predictive of worse treatment outcomes (e.g., Eisler et al. 2000, 2007; Rienecke et al. 2016). Interestingly, perceived criticism did not predict reductions in dietary restraint, which may be due to differences in the construct measured (i.e., change in restraint vs. change in BMI Centile). Importantly, measures of dietary restraint appear to capture intention to restrict and cognitive restraint, which may be more likely to lead to overeating and weight gain in the long term rather than restricted intake or undereating (van Strien et al. 2014), which is supported (albeit cross sectionally) by the positive correlation between BMI centile and restraint in the current sample. These findings suggest that perceived criticism may present a barrier to food intake and weight gain among young people with EDs, but less of a barrier to addressing cognitive dietary restraint.

Taken together, these results have important implications regarding the mechanisms through which Strong Foundations may be effective, and who it is most suitable for. Future research may look to dismantling studies to further understand the contributions of components of Strong Foundations to observed outcomes (i.e., seminar sessions vs. medical monitoring appointments), which cannot be made from the current study. The findings related to parental self‐efficacy and expressed emotion continue to support the notion that family factors are important to consider in child and adolescent ED treatment, and they can be both a pathway (improved self‐efficacy) and a barrier (high expressed emotion) to recovery. It seems important that treatments continue to focus and refine even more potent ways of improving parental self‐efficacy, whilst also exploring ways of better meeting the needs and addressing expressed emotion when this presents a barrier to treatment. Future revisions and developments of new focused, program‐led interventions may benefit from focusing on incorporating strategies targeting those families with high expressed criticism, in line with recent evidence showing that selective intervention improves treatment outcomes (Aarnio‐Peterson et al. 2024). Alternative interventions may circumvent this barrier; for example, interventions that focus more on engaging the young person in initiating change themselves (as in CBT‐E), rather than relying on family‐focused approaches, may be better suited to families with high expressed emotion. Given CBT‐E has existing evidence for self‐help and focused versions (Allen et al. 2024), continuing to adapt this framework into more easily accessed forms may increase early, effective interventions.

There are several limitations to the current study that could be addressed through future research. There was no comparison group utilized, which prevents attribution of the effects found to the intervention and not another variable (e.g., time). No follow‐up data were collected, which limits the identification of mechanisms or predictors of treatment effects that may have taken longer to establish (e.g., changes to BMI), the sustainability of treatment effects, and/or impact on subsequent interventions. As the study design measured within‐person observations at the same time points, we cannot infer causality in the proposed mediation model (i.e., it cannot be said that changes to parental self‐efficacy caused changes in youth restraint). The dataset contained significant missing data at post‐treatment, which is reflective of the real‐world setting with limited research administration support. However, this limits the robustness of the results as effects may be over‐or under‐estimated. As discussed, the high correlation between restraint and food‐related obsessions and rituals suggests significant overlap between constructs, which limits the findings related to these variables. Overall, the current results explained only a modest proportion of variance, suggesting other factors are likely contributing to treatment outcomes. The current sample consisted mostly of young females with AN or AAN, which limits the generalizability of the results to young people with other EDs. Similarly, other measures of diversity (e.g., ethnicity, sexuality, gender identity) have been incorporated into routine data collection at this site; however, this change predated data collection for the current study. Future research should continue to be mindful of being inclusive of minority populations when considering research methodology and data collection. Collectively, while more methodologically rigorous studies (e.g., randomized controlled trials) are still needed, these findings offer useful preliminary insights that can inform hypotheses and contribute to shaping future research. A strength of the current study included its real‐world setting in a public community mental health service, which supports the external validity of the results.

## 5 Conclusions

Programme‐led and focused intervention have the potential to overcome some of the barriers that young people with ED face in accessing treatment, with evidence for this emerging field appearing promising. We found that the effect of Strong Foundations on reductions in dietary restraint was mediated by improvements to parental self‐efficacy, and that high perceived criticism was a barrier to weight gain among young people with ED. Findings support the importance of empowering families to not only help their children recover from an ED, but also to manage expressed criticism in this often difficult and challenging journey. Future research could investigate moderators between treatment (e.g., comparing family focussed vs. individual treatments), replicate the current results in more diverse populations, and explore treatment adaptations aimed at increasing the potency of such interventions.

## Author Contributions

**Daniel Wilson:** conceptualization, investigation, funding acquisition, writing – original draft, methodology, validation, formal analysis. **Renata A. Mendes:** data curation, project administration, writing – review and editing, methodology. **Natalie J. Loxton:** writing – review and editing, supervision, methodology.

## Ethics Statement

The project was approved by the Children's Health Queensland Hospital and Health Service Human Research Ethics Committee (EC00175) HREC/20/QCHQ/67708. Informed consent was obtained from parents/legal guardians as per ethical approval.

## Consent

The authors have nothing to report.

## Conflicts of Interest

The authors declare no conflicts of interest.

## Supporting information


**Data S1.** eat24516‐sup‐0001‐Tables.docx.

## Data Availability

The data that support the findings of this study are available on request from the corresponding author. The data are not publicly available due to privacy or ethical restrictions.

## References

[eat24516-bib-0001] Aarnio‐Peterson, C. M. , D. Le Grange , C. A. Mara , et al. 2024. “Emotion Coaching Skills as an Augmentation to Family‐Based Therapy for Adolescents With Anorexia Nervosa: A Pilot Effectiveness Study With Families With High Expressed Emotion.” International Journal of Eating Disorders 57, no. 3: 682–694. 38318997 10.1002/eat.24149 PMC10947854

[eat24516-bib-0002] Allen, K. L. , L. Courtney , P. Croft , et al. 2024. “Programme‐Led and Focused Interventions for Recent Onset Binge/Purge Eating Disorders: Use and Outcomes in the First Episode Rapid Early Intervention for Eating Disorders (FREED) Network.” International Journal of Eating Disorders 58, no. 2: 389–399. 10.1002/eat.24343. 39614775 PMC11861885

[eat24516-bib-0003] Brown, G. , J. Birley , and J. Wing . 1972. “Influence of Family Life on the Course of Schizophrenic Disorders: A Replication.” British Journal of Psychiatry 121: 241–258. 10.1192/bjp.121.3.241 5073778

[eat24516-bib-0004] Carter, O. , L. Pannekoek , A. Fursland , K. L. Allen , A. M. Lampard , and S. M. Byrne . 2012. “Increased Wait‐List Time Predicts Dropout From Outpatient Enhanced Cognitive Behaviour Therapy (CBT‐E) for Eating Disorders.” Behaviour Research and Therapy 50, no. 7–8: 487–492. 22659158 10.1016/j.brat.2012.03.003

[eat24516-bib-0005] Collaborators, G. M. D. 2022. “Global, Regional, and National Burden of 12 Mental Disorders in 204 Countries and Territories, 1990‐2019: A Systematic Analysis for the Global Burden of Disease Study 2019.” Lancet Psychiatry 9, no. 2: 137–150. 10.1016/S2215-0366(21)00395-3. 35026139 PMC8776563

[eat24516-bib-0006] Couturier, J. , S. Sami , M. Nicula , et al. 2023. “Examining the Feasibility of a Parental Self‐Help Intervention for Families Awaiting Pediatric Eating Disorder Services.” International Journal of Eating Disorders 56, no. 1: 276–281. 36285643 10.1002/eat.23837

[eat24516-bib-0007] Davey, E. , K. Allen , S. D. Bennett , et al. 2023. “Improving Programme‐Led and Focused Interventions for Eating Disorders: An Experts' Consensus Statement—A UK Perspective.” European Eating Disorders Review 31, no. 5: 577–595. 10.1002/erv.2981. 37218053 PMC10947440

[eat24516-bib-0008] Eisler, I. , C. Dare , M. Hodes , G. Russell , E. Dodge , and D. Le Grange . 2000. “Family Therapy for Adolescent Anorexia Nervosa: The Results of a Controlled Comparison of Two Family Interventions.” Journal of Child Psychology and Psychiatry 41, no. 6: 727–736. 11039685

[eat24516-bib-0009] Eisler, I. , M. Simic , G. F. Russell , and C. Dare . 2007. “A Randomised Controlled Treatment Trial of Two Forms of Family Therapy in Adolescent Anorexia Nervosa: A Five‐Year Follow‐Up.” Journal of Child Psychology and Psychiatry 48, no. 6: 552–560. 17537071 10.1111/j.1469-7610.2007.01726.x

[eat24516-bib-0010] Fairburn, C. G. , and S. J. Beglin . 1994. “Assessment of Eating Disorders: Interview or Self‐Report Questionnaire?” International Journal of Eating Disorders 16, no. 4: 363–370. 7866415

[eat24516-bib-0011] Fitzpatrick, M. E. , and T. Weltzin . 2014. “Motivation for Change as a Predictor of Eating Disorder Treatment Outcomes Using a Brief Self‐Report YBC‐EDS in a Residential Eating Disorder Population.” Eating Behaviors 15, no. 3: 375–378. 10.1016/j.eatbeh.2014.04.007. 25064284

[eat24516-bib-0012] Forman, S. F. , L. F. Grodin , D. A. Graham , et al. 2011. “An Eleven Site National Quality Improvement Evaluation of Adolescent Medicine‐Based Eating Disorder Programs: Predictors of Weight Outcomes at One Year and Risk Adjustment Analyses.” Journal of Adolescent Health 49, no. 6: 594–600. 10.1016/j.jadohealth.2011.04.023 22098769

[eat24516-bib-0013] Gorrell, S. , C. Byrne , P. Trojanowski , S. Fischer , and D. Le Grange . 2022. “A Scoping Review of Non‐Specific Predictors, Moderators, and Mediators of Family‐Based Treatment for Adolescent Anorexia and Bulimia Nervosa: A Summary of the Current Research Findings.” Eating and Weight Disorders 27, no. 6: 1971–1990. 10.1007/s40519-022-01367-w. 35092554 PMC9872820

[eat24516-bib-0014] Hamadi, L. , and J. Holliday . 2020. “Moderators and Mediators of Outcome in Treatments for Anorexia Nervosa and Bulimia Nervosa in Adolescents: A Systematic Review of Randomized Controlled Trials.” International Journal of Eating Disorders 53, no. 1: 3–19. 31506978 10.1002/eat.23159

[eat24516-bib-0015] Judd, C. M. , D. A. Kenny , and G. H. McClelland . 2001. “Estimating and Testing Mediation and Moderation in Within‐Subject Designs.” Psychological Methods 6, no. 2: 115–134. 10.1037/1082-989x.6.2.115. 11411437

[eat24516-bib-0016] Kazdin, A. E. , E. E. Fitzsimmons‐Craft , and D. E. Wilfley . 2017. “Addressing Critical Gaps in the Treatment of Eating Disorders.” International Journal of Eating Disorders 50, no. 3: 170–189. 28102908 10.1002/eat.22670 PMC6169314

[eat24516-bib-0017] Le Grange, D. , E. C. Accurso , J. Lock , S. Agras , and S. W. Bryson . 2014. “Early Weight Gain Predicts Outcome in Two Treatments for Adolescent Anorexia Nervosa.” International Journal of Eating Disorders 47, no. 2: 124–129. 24190844 10.1002/eat.22221 PMC4341963

[eat24516-bib-0020] Le Grange, D. , J. Lock , W. S. Agras , et al. 2012. “Moderators and Mediators of Remission in Family‐Based Treatment and Adolescent Focused Therapy for Anorexia Nervosa.” Behaviour Research and Therapy 50, no. 2: 85–92. 22172564 10.1016/j.brat.2011.11.003 PMC3260378

[eat24516-bib-0019] Le Grange, D. , J. Lock , W. S. Agras , A. Moye , S. W. Bryson , and B. Jo . 2015. “Randomized Clinical Trial of Family‐Based Treatment and Cognitive‐Behavioral Therapy for Adolescent Bulimia Nervosa.” Journal of the American Academy of Child and Adolescent Psychiatry 54, no. 11: 886–894. 10.1016/j.jaac.2015.08.008. 26506579 PMC4624104

[eat24516-bib-0021] Lock, J. , W. Agras , S. Bryson , and H. Kraemer . 2005. “A Comparison of Short‐ and Long‐Term Family Therapy for Adolescent Anorexia Nervosa.” Journal of the American Academy of Child and Adolescent Psychiatry 44, no. 7: 632–639. 10.1097/01.chi.0000161647.82775.0a. 15968231

[eat24516-bib-0022] Lock, J. , and D. Le Grange . 2012. Treatment Manual for Anorexia Nervosa: A Family‐Based Approach. 2nd ed. Guilford Press.

[eat24516-bib-0023] Lock, J. , and D. Le Grange . 2019. “Family‐Based Treatment: Where Are We and Where Should We Be Going to Improve Recovery in Child and Adolescent Eating Disorders.” International Journal of Eating Disorders 52, no. 4: 481–487. 10.1002/eat.22980. 30520532

[eat24516-bib-0024] Montoya, A. K. , and A. F. Hayes . 2017. “Two‐Condition Within‐Participant Statistical Mediation Analysis: A Path‐Analytic Framework.” Psychological Methods 22, no. 1: 6–27. 10.1037/met0000086. 27362267

[eat24516-bib-0025] Rienecke, R. D. , E. C. Accurso , J. Lock , and D. Le Grange . 2016. “Expressed Emotion, Family Functioning, and Treatment Outcome for Adolescents With Anorexia Nervosa.” European Eating Disorders Review 24, no. 1: 43–51. 10.1002/erv.2389. 26201083 PMC4962527

[eat24516-bib-0026] Rienecke, R. D. , and D. Le Grange . 2022. “The Five Tenets of Family‐Based Treatment for Adolescent Eating Disorders.” Journal of Eating Disorders 10, no. 1: 60. 10.1186/s40337-022-00585-y. 35505444 PMC9066936

[eat24516-bib-0027] Sawyer, S. M. , M. Whitelaw , D. Le Grange , M. Yeo , and E. K. Hughes . 2016. “Physical and Psychological Morbidity in Adolescents With Atypical Anorexia Nervosa.” Pediatrics 137, no. 4: e20154080. 10.1542/peds.2015-4080. 27025958

[eat24516-bib-0028] Sidari, M. J. , D. Wilson , S. Catania , et al. 2025. “Pre‐Treatment Specialist Interventions Improve Parents' Self‐Efficacy and Their Children's Eating Disorder Symptomology Before Commencing Outpatient Treatment.” European Eating Disorders Review. 10.1002/erv.3211. PMC12547362 40411793

[eat24516-bib-0029] Spettigue, W. , D. Maras , N. Obeid , et al. 2015. “A Psycho‐Education Intervention for Parents of Adolescents With Eating Disorders: A Randomized Controlled Trial.” Eating Disorders 23, no. 1: 60–75. 25090010 10.1080/10640266.2014.940790

[eat24516-bib-0030] Turner, H. , R. Bryant‐Waugh , and E. Marshall . 2015. “The Impact of Early Symptom Change and Therapeutic Alliance on Treatment Outcome in Cognitive‐Behavioural Therapy for Eating Disorders.” Behaviour Research and Therapy 73: 165–169. 26334452 10.1016/j.brat.2015.08.006

[eat24516-bib-0031] van Strien, T. , C. P. Herman , and M. W. Verheijden . 2014. “Dietary Restraint and Body Mass Change. A 3‐Year Follow Up Study in a Representative Dutch Sample.” Appetite 76: 44–49. 10.1016/j.appet.2014.01.015. 24480668

[eat24516-bib-0032] Viljoen, D. , E. King , S. Harris , et al. 2024. “The Alarms Should No Longer Be Ignored: Survey of the Demand, Capacity and Provision of Adult Community Eating Disorder Services in England and Scotland Before COVID‐19.” BJPsych Bulletin 48, no. 4: 212–220. 10.1192/bjb.2023.57. PMC11543318 37525957

[eat24516-bib-0033] Von Holle, A. , A. Poyastro Pinheiro , L. M. Thornton , et al. 2008. “Temporal Patterns of Recovery Across Eating Disorder Subtypes.” Australian and New Zealand Journal of Psychiatry 42, no. 2: 108–117. 18197505 10.1080/00048670701787610

[eat24516-bib-0034] Wade, T. , S. Byrne , A. Fursland , et al. 2022. “Is Guided Self‐Help Family‐Based Treatment for Parents of Adolescents With Anorexia Nervosa on Treatment Waitlists Feasible? A Pilot Trial.” International Journal of Eating Disorders 55, no. 6: 832–837. 35470910 10.1002/eat.23720

[eat24516-bib-0035] Wilson, D. , T. Withington , G. Krishnamoorthy , M. Dalton , R. A. Mendes , and N. J. Loxton . 2025. “A Comparison of Psychiatric Comorbid Symptomology Between Adolescents With Restrictive/Avoidant Food Intake Disorder, Anorexia Nervosa and Atypical Anorexia Nervosa.” European Eating Disorders Review. 10.1002/erv.70014. PMC12694686 40682796

[eat24516-bib-0036] Zanna, V. , M. Criscuolo , A. Mereu , et al. 2021. “Restrictive Eating Disorders in Children and Adolescents: A Comparison Between Clinical and Psychopathological Profiles.” Eating and Weight Disorders: EWD 26, no. 5: 1491–1501. 10.1007/s40519-020-00962-z. 32720247

