# Supplementary material for: Predictors and Mediators of Outcome of a Focussed, Programme Led Intervention for Young People With Eating Disorders
Source: Int J Eat Disord. 2025 Aug 11;58(11):2138–46. doi: 10.1002/eat.24516 (PMC12605778; doi:10.1002/eat.24516)
Supplement: Supplementary file 1 — Data S1. eat24516‐sup‐0001‐Tables.docx. [file EAT-58-2138-s001.docx]

| Measure (range) | Pre mean (SD) | | Post mean (SD) | T | *p* | *d* | 95% CI of d | |
| --- | --- | --- | --- | --- | --- | --- | --- | --- |
|  |  |  | |  |  |  | Lower | Upper |
| BMI Centile | 36.87 (28.48) | 44.48 (27.33) | | -7.02 (159) | <.001 | -.54 | -.70 | -.38 |
| Restraint (0-6) | 3.37 (1.99) | 2.68 (1.98) | | 4.81 (163) | <.001 | .38 | .22 | .53 |
| EDE-Q – Global (0-6) | 3.72 (1.65) | 3.18 (1.78) | | 5.25 (163) | <.001 | .41 | .25 | .57 |
| Self-efficacy (Primary) (5-35) | 18.98 (3.68) | 20.62 (3.91) | | -6.60 (164) | <.001 | -.51 | -.68 | -.35 |
| Self-efficacy (female) (5-35) | 18.89 (3.68) | 20.66 (3.93) | | -6.58 (146) | <.001 | -.54 | -.72 | -.37 |
| Self-efficacy (male) (5-35) | 19.59 (3.06) | 20.93 (3.25) | | -4.96 (93) | <.001 | -.50 | -.72 | -.29 |

Table S1. Pre and post Strong Foundations means, standards deviations, and effect sizes.

Note:. ED obsessions and rituals = Yale Brown Cornell Eating Disorders Scale; Perceived criticism = Brief Dyadic Scale of Expressed Emotion - Perceived Criticism subscale; Self-efficacy = Parent versus Anorexia scale; EDE-Q – Global = Eating Disorders Examination Questionnaire – Global subscale; *d* = Cohens’ d; CI = confidence intervals.

**Mediation analysis using EDE-Q.** The results of the mediation model showed that changes to parental self-efficacy were found to significantly mediate the effects of time (intervention) on the reduction of EDE-Q scores (indirect effect = .12, SE = .06, 95%CI: .02; .26). The total model explained 4% of the variance in changes to restraint scores (*R^2^* = .04, *F* (2,159) = 3.23, *p* = .04).

**Regression analysis using EDE-Q.** The multiple regression analysis was conducted with ED obsessions and rituals and perceived criticism as predictors of change in EDE-Q. The model was not statistically significant, *R* = .18, *R^2^* = .03, F(2, 119) = 1.91, *p* = .15, and neither variables were significant predictors, as shown in Table S2.

Table S2. Effects of ED obsession and rituals, and Perceived Criticism on changes to EDE-Q across Strong Foundations.

| Criterion | Variable | B | SE | p | 95%CI lower | 95%CI upper | sr^2^ |
| --- | --- | --- | --- | --- | --- | --- | --- |
| Change in EDE-Q - Global |  |  |  |  |  |  |  |
|  | ED obsessions and rituals | .03 | .02 | .09 | -.01 | .06 | .02 |
|  | Perceived criticism | -.02 | .01 | .20 | -.05 | .01 | .01 |
|  |  |  |  |  |  |  |  |

Note: Unstandardised coefficients are shown. CI = confidence interval. ED obsessions and rituals = Yale Brown Cornell Eating Disorders Scale; Perceived criticism = Brief Dyadic Scale of Expressed Emotion - Perceived Criticism subscale; EDE-Q – Global = Eating Disorders Examination Questionnaire – Global subscale; Change in EDE-Q = pre score-post score, meaning that a higher score indicates greater reductions in EDE-Q scores across the treatment. As such, positive coefficients predict a greater response to treatment whereas negative coefficients predict a lesser response to treatment.
